# Benchmarking the nutrition-related commitments and practices of major French food companies

**DOI:** 10.1186/s12889-022-13780-y

**Published:** 2022-07-28

**Authors:** Iris Van Dam, Stefanie Vandevijvere

**Affiliations:** 1 Sciensano, Service of Lifestyle and chronic diseases, Brussels, Belgium; 2 Université Paris-Saclay, INRAE, UR ALISS, 94205 Ivry-sur-Seine, France

**Keywords:** Business impact assessment, Food industry, Nutritional quality, Food supply, Nutrient profile, Accountability

## Abstract

**Background:**

This study benchmarked and quantitatively assessed the transparency, specificity and comprehensiveness of nutrition-related commitments and related practices of the major companies within the French food industry.

**Methods:**

To evaluate the nutrition-related commitments and practices across policy domains such as product reformulation, labelling, marketing, and accessibility, the ‘Business Impact Assessment on Obesity and population-level nutrition’ (BIA-Obesity) was applied. A total of 33 French food companies were selected using Euromonitor 2018 market share data, including major packaged food and non-alcoholic beverage manufacturers (*N =* 20), quick-service restaurants (*N =* 7), and supermarkets (*N =* 6). During 2019-2020 the publicly available commitments were collected for each company, scored according to the BIA-Obesity, and company representatives were provided with the opportunity to complete and verify the collected data. The following performance metrics were included to assess company practices: the median Nutri-Score of product portfolios, the proportion of products with Nutri-Score A or B, the percentage of products (not-)permitted to be marketed to children according to the World Health Organisation Europe nutrient profile model and the proportion of ultra-processed food products as determined by the NOVA-classification. In addition supermarket flyers were collected over a 6-months period to assess the healthiness of product promotions. Correlations between commitments and performance metrics were assessed applying the Spearman’s rank correlation coefficient.

**Results:**

Among the selected food companies, 13 companies verified and completed the publicly available data (response rate = 39%). Overall BIA-Obesity scores for company commitments varied between 2 and 74% with a median score of 28%. Scores for packaged food and non-alcoholic beverage manufacturers were higher than those for supermarkets and quick-service restaurants. The median proportion of foods with Nutri-Score A or B within product portfolios was 38% (range = 1-95%), while the median proportion of non-permitted products was 84% (range = 7-100%) and the median proportion of ultra-processed food products 63% (range = 5-100%). Stronger company commitments did not translate into better performance metrics.

**Conclusions:**

There is room for significant improvement of both company commitments and performance. Current food industry action does not meet recommended best practices. The French government is urged to regulate food industry practices to create healthier food environments.

**Supplementary Information:**

The online version contains supplementary material available at 10.1186/s12889-022-13780-y.

## Background

In France about two out of five adults and one in seven adolescents have a body mass index (BMI) above 25 kg/m^2^ and as such can be considered to live with overweight or obesity [1]. Both overweight and obesity significantly increase the risk of non-communicable diseases (NCDs) [2, 3], which are major public health problems in France. A high BMI, as well as unhealthy diets are among the top risk factors driving death and disability [4]. Nonetheless, French people consume about one third of their energy from ultra-processed food products [5]. High consumption of such products has been associated with weight gain, overweight and even increased mortality [6–9].

The high consumption of such food products is driven by the current policy environment, which allows the food industry to affect food environments without taking into account the vast health impact [10–12]. Most food companies have commitments in place to improve the healthiness of food environments through voluntary marketing codes, selected reformulation targets and labelling initiatives. However, such voluntary codes often fall short of recommended best practices [13–17]. As a result it becomes of utmost importance to monitor and evaluate food company commitments as well as their practices to ensure that commitments translate into real-world improvement of marketing practices, healthiness of product portfolios, front-of-pack (FOP) labelling practices and increased accessibility of healthier products across different settings [18, 19]. Moreover, improving population nutrition is crucial in achieving the United Nations Sustainable Development Goals (SDGs) [20].

While food companies make individual commitments as part of their corporate social responsibility, there are also government-led initiatives in place in France. The most well-known policy is the Nutri-Score, the government endorsed FOP labelling system that was introduced in France in 2017 and classifies products in five product categories (A being the most healthy to E being the least healthy category) based on the nutrient composition per 100 g/ml [21, 22]. In terms of reformulation, companies have been encouraged to reduce nutrients of concern such as salt, sugar, fat and trans-fat across product portfolios by the ‘Voluntary Commitment Charter for Nutritional Progress’ (*‘La charte d’engagement volontaires de progrès nutritionnel’*) [23]. Through this charter, voluntary company commitments to improve the nutritional quality of products are validated by public authorities [23]. In contrast to several other countries, there is no overarching industry pledge in place in France to limit the marketing of unhealthy food products to children [24–26]. Companies can however sign up to the European wide initiative, the EU-Pledge, through which commitments are made to not market products to children below the age of 12 years that do not meet the set out nutrition criteria [27, 28]. Still, these nutrition criteria have been under scrutiny for not adequately protecting children from unhealthy food marketing [29, 30]. An alternative model, the World Health Organisation Regional Office for Europe nutrient profile model (WHO-model), with much stricter nutrition criteria has however been developed to overcome the aforementioned shortcoming [30, 31].

This study set out to, for the first time, benchmark and quantitatively assess the commitments and practices related to obesity prevention and population nutrition of the largest French food companies. The study included four industry sectors: packaged food manufacturers, non-alcoholic beverage manufacturers, supermarkets and quick-service restaurants. The objective was to highlight where French food companies are demonstrating leadership in relation to obesity prevention and nutrition, and to identify areas for improvement. In addition, this study aimed to assess whether stronger nutrition-related commitments translated into stronger practices and performance.

## Methods

To assess food industry commitments and practices, the ‘Business Impact Assessment on Obesity and population-level nutrition’ (BIA-Obesity) was applied, as developed by the International Network for Food and Obesity/Non-communicable Diseases Research, Monitoring and Action Support (INFORMAS) and previously described in detail by Sacks et al. [10, 18]. The tool assesses the transparency, comprehensiveness and specificity of commitments as well as practices across six domains, namely: ‘Corporate nutrition strategy’, ‘Product formulation’, ‘Nutrition labelling’, ‘Product and brand promotion’, ‘Product accessibility’ and ‘Relationships with other organisations’ [18].

All indicators within these domains relate to commitments that go beyond legislative requirements. As a result, indicators and scoring criteria need to be adapted to the local context prior to implementation of the tool. Indicators related to the on-pack disclosure of the ingredients list and nutritional declaration were removed as this is regulated by the European Union [32]. As it is not common in France for supermarkets to have in-store restaurants, indicators relating to menu-labelling were removed for this food industry. Furthermore, non-alcoholic beverages containing added sugars or sweeteners in France are subject to a tax [33]. Consequently, commitments to increase prices of sugary beverages compared to healthier drinks were not taken into account. Since the provision of unlimited refills was banned in France in 2017 [34] the indicator relating to commitments of quick-service restaurants to not provide free refills was removed. Lastly, the indicator regarding the publication of political donations was removed as in France legal persons (including, and in particular, companies) are not authorized to pay any donation or any benefit in kind to political parties [35]. The remaining indicators were adapted to suit the French regulatory environment and take into account relevant industry pledges and voluntary government-led initiatives (i.e. Nutri-Score).

This study was approved by the Human Ethics Committee of the University of Ghent (number: 2019/0780).

### Selection of food companies

Food companies with a combined market share of over 34% among packaged food manufacturers (35%), non-alcoholic beverage manufacturers (52%), supermarkets (48%) and quick-service restaurants (50%) were selected using French Euromonitor 2018 market share data (Table 1) [36]. For packaged food manufacturers, an additional selection was conducted based on companies’ market share within specific food categories to ensure that the most prominent companies per food category were covered by the selection (*‘Breakfast cereals’, ‘Baked goods’ ‘Confectionery’, ‘Ice-cream and frozen desserts’, ‘Processed Fruit and Vegetables’, ‘Processed Meat and Seafood’, ‘Sweet biscuits and cereal bars’, ‘Drinking milk products’, ‘Yoghurts’, ‘Savoury snacks’ and ‘Ready meals’*). Three additional companies were included based on this extra selection (Kellogg’s, Barilla and Bonduelle).

**Table 1:** The market shares per food industry as determined by Euromonitor and most sold product categories of companies included in the study (France, Euromonitor, 2018)

Packaged food manufacturers		
Companies	Market share (%)	Most sold (own-brand) product categories
*Lactalis*	3.4	Dairy
*Mondelēz*	2.9	Bread & bakery products, Confectionary, Savoury snack foods
*Nestlé*	2.6	Dairy, Confectionary, Non-alcoholic beverages
*Ferrero*	2.1	Confectionary, Bread & bakery products, Cereal & grain products
*Fleury Michon*	1.9	Meat & fish products, Convenience foods
*Danone*	1.6	Dairy, Non-alcoholic beverages
*Unilever*	1.3	Dairy, Sauces, Convenience foods
*Savencia*	1.3	Dairy, Confectionary, Meat & fish products
*Bel*	1.2	Fruit & vegetable products, Dairy
*Panzani*	1.0	Cereal & grain products, Convenience foods, Sauces
*Barilla* ^*1*^	0.9	Bread & bakery products, Cereal & grain products, Sauces
*Bonduelle* ^*2*^	0.6	Fruit & vegetable products, Convenience foods
*Kellogg’s* ^*3*^	0.5	Cereal & grain products, Savoury snack foods
*William Saurin*	0.3	Convenience foods, Meat & fish products
*N = 14*	***21.6*** ^***4***^	
**Non-alcoholic beverage manufacturers**		
*Coca-Cola*	17.2	Non-alcoholic beverages
*PepsiCo*	8.8	Non-alcoholic beverages, Savoury snack foods, Cereal & grain products
*Orangina Suntory*	7.6	Non-alcoholic beverages
*Eckes-Granini*	3.9	Non-alcoholic beverages
*Fruité Entreprises*	4.0	Non-alcoholic beverages
*Andros*	2.0	Fruit & vegetable products, Dairy, Bread & bakery products, Non-alcoholic beverages
*N = 6*	***43.5*** ^***5***^	
**Supermarkets**		
*E. Leclerc*	11.1	Dairy, Fruit & vegetable products, Meat & fish products
*Intermarché*	9.8	Dairy, Fruit & vegetable products, Bread & bakery products
*Carrefour*	8.8	Dairy, Fruit & vegetable products, Meat & fish products
*Auchan*	8.2	Meat & fish products, Fruit & vegetable products, Dairy
*Super U*	5.2	Meat & fish products, Fruit & vegetable products, Dairy
*Lidl*	4.4	
*N = 6*	***47.5***	
**SupermarketsQuick-service restaurants**		
*McDonald’s*	32.2	Burgers
*KFC*	4.1	Burgers
*Quick*	3.9	Burgers
*Burger King*	2.9	Burgers
*Paul*	2.6	Bread & bakery products, Convenience foods
*La Brioche Dorée*	2.1	Bread & bakery products, Convenience foods
*Domino’s Pizza*	1.9	Pizza
*N = 7*	***49.7***	

1: The largest market share within the Euromonitor food category ‘Baked goods’

2: The largest market share within the Euromonitor food category ‘Processed Fruit and Vegetables’

3: The largest market share within the Euromonitor food category ‘Breakfast cereals’

4 and 5: Excluding the supermarkets as food and beverage manufacturers (market share foods: 13.2%; market share beverages: 8.2%)

### Data collection and analyses

#### Nutrition-related commitments

Publicly available commitments and policies were collected between June 2019 and December 2020. Relevant information was collected from company websites, company reports, brand websites and relevant industry pledges and initiatives. Per selected company, screenshots were taken of relevant webpages and relevant documents were downloaded.

Subsequently, the information was entered in an Excel spreadsheet per BIA-Obesity indicator. A report summarizing the collected information as well as the preliminary scoring was compiled per company. Company representatives were contacted via various channels, including meetings with industry associations (ANIA and L’Alliance 7), phone call inquiries, contact information on company/brand websites and LinkedIn. Companies willing to verify and complete the collected data were sent the summary reports after signing a written informed consent. For all additional information they provided some kind of evidence was required. Upon request companies could sign non-disclosure agreements prior to sharing sensitive internal documents. For companies that refused participation or failed to share feedback in time, the assessment was based solely on publicly available information. Supermarkets were assessed as both retailers and food manufacturers (the latter for own-brand products).

The nutrition-related commitments were scored in Excel. Supplementary file 1 provides examples of how scores were assigned for BIA-Obesity indicators. All company commitments were scored by IVD and two companies per food industry (a total of eight companies) were blindly re-scored by YZ. Discrepancies were discussed until an agreement was obtained. The final BIA-Obesity scores per domain were weighted as recommended by INFORMAS (Supplementary file 2) [18].

Median scores (range and interquartile range IQR), overall and per BIA-Obesity domain, were calculated for each food industry and across food industries. For companies that verified and completed the publicly available information, median scores before and after their participation were calculated. A one-tailed Wilcoxon signed-rank test was conducted to compare scores before and after participation. The Wilcoxon signed-rank test was applied as the test assessed changes in a dependent outcome variable before and after companies had the opportunity to provide additional information. It was opted for a one-tailed test as companies could only improve their scoring by sharing extra information in addition to the publicly available evidence. A two-tailed Wilcoxon rank-sum test was used to compare scores of two independent groups, namely companies that engaged with the process and those that did not engage. Both tests are non-parametric tests.

#### Practices

For some of the BIA-Obesity policy domains, a set of key performance indicators was selected to assess company practices on population nutrition. The selected indicators, as well as the sources where the data were derived from and the years, are presented below in Table 2. For the domains on ‘Corporate nutrition strategy’ and ‘Relationships with other organisations’, no performance indicators (such as an assessment of companies’ corporate political activities) were included due to a lack of time and resources available to collect data within these domains. For the domains ‘Nutrition labelling’ and ‘Product accessibility’ no performance data were available at the time of assessment. For the other BIA-Obesity domains, specific indicators were included, dependent on data availability and feasibility of the assessment. An overview of the different performance indicators can be found in Table 2.

**Table 2:** An overview of the performance indicators per food industry and ‘Business Impact assessment on Obesity and Population Nutrition’ (BIA-Obesity) domain. The data source and the year of data collection are specified per indicator

Food Industry	BIA-Obesity Domain	Performance indicators	Data sources	Years
Food and beverage manufacturers	**Product formulation**	*For full product portfolio:* ✓ Median Nutri-Score ✓ % of products with Nutri-Score A and B ✓ % of products with Nutri-Score D and E ✓ % of products that are ultra-processed	Open Food Facts data France^1^	2018
	**Product and brand promotion**	*For full product portfolio:* ✓ % of products not-permitted to be marketed to children according to the World Health Organisation Regional Office for Europe nutrient profile model (WHO-Model)	Open Food Facts data France^1^	2018
Supermarkets	**Product formulation**	*For full own-brand product portfolio:* ✓ Median Nutri-Score ✓ % of Nutri-Score A and B ✓ % of Nutri-Score D and E ✓ % of products that are ultra-processed	Open Food Facts data France^1^	2018
	**Product and brand promotion**	*For full own-brand product portfolio:* ✓ % of products not permitted to be marketed to children according to the WHO-Model *For all food products:* ✓ % of promotions for foods that are ultra-processed ✓ % of promotions for fresh fruit and vegetables ✓ % of promotions with promotional characters ✓ % of promotions with discounts ✓ % of promotions with incentive offers	Open Food Facts data France^1^ Supermarket circulars	2018 October 2019 – March 2020
Quick-service restaurants	**Product formulation**	*For online product portfolio:* ✓ Median Nutri-Score ✓ % of products with Nutri-Score A and B ✓ % of products with Nutri-Score D and E	Company websites	2019 ^2^
	**Product and brand promotion**	*For online product portfolio:* ✓ % of products not-permitted to be marketed to children according to the WHO-Model	Company websites	2019 ^2^

^1^Verified using Mintel GNPD (Global New Products Database) data or nutritional values from brand or supermarket websites

^2^2018 for KFC. No data available for Brioche Dorée and Quick

##### Product formulation

For packaged food and non-alcoholic beverage manufacturers and supermarkets (own-brand products), the healthiness of the complete product portfolios was analysed using Open Food Facts data for France in 2018. As Open Food Facts cannot guarantee the accuracy and completeness of the data, the nutritional data of all products that could be found on Mintel GNPD (Global New Products Database), on brand websites or supermarket websites were verified using the aforementioned sources. Duplication of products was avoided by ensuring that each barcode appeared only once.

For quick-service restaurants, the nutritional information per 100 g was obtained from the national brand websites in 2019, where possible (Burger King, Domino’s Pizza, McDonald’s and Paul). For KFC no nutritional information was available per 100 g and no portion sizes were specified on the national website, so an online table with nutritional information from 2018 was used. On the website of Brioche Dorée and Quick no nutritional information was available per 100 g and portion sizes were not defined. As a result, the product portfolios of Brioche Dorée and Quick could not be analysed.

The healthiness of the entire portfolios of all selected food companies was analysed using the Nutri-Score, which is the official front-of-pack labelling system in place in France since March 2017 [21]. The proportion of products with Nutri-Score A, B, C, D and E was determined, as well as the median Nutri-Score across the company’s portfolio or menu. When calculating the Nutri-Score for non-alcoholic beverages, it was assumed that no juices had a fruit and vegetable content above 40% as the data sources and product ingredient lists did not allow for a distinction to be made between the fruit and vegetable content of different juices. To check the viability of this assumption, a Pearson correlation coefficient was calculated between the Nutri-Score available through Open Food Facts and the calculated Nutri-Score for non-alcoholic beverages. A strong correlation was observed between both Nutri-scores (*R =* 0.84, *p* < 0.0001). In addition, a correlation between the Open Food Facts Nutri-Score and the calculated Nutri-Score was also conducted for the entire dataset. A very strong correlation was observed between the calculated Nutri-Score and the Nutri-Score displayed within Open Food Facts (*R =* 0.98, *p* < 0.0001).

The company’s portfolios were also analysed in relation to the proportion of ultra-processed foods (according to the NOVA classification [37]). The NOVA-classification distinguishes products based on their level of processing (unprocessed or minimally processed foods, processed culinary ingredients, processed foods and ultra-processed foods) [37]. The proportion of products within portfolios that are ultra-processed (NOVA) as well as the median Nutri-Score and the proportion of products with Nutri-Score ‘A and B’ and ‘D and E’, were examined by company. The results were reported as a proportion of products with Nutri-Score ‘A and B’ and ‘D and E’ as this was considered to reflect the healthiness of companies’ overall product portfolios. The proportion of products with Nutri-Score ‘A and B’ was deemed to represent healthier alternatives within the product portfolio while the proportion with Nutri-Score ‘D and E’ was considered to signify less healthy products.

##### Product and brand promotion

To assess the proportion of products within company portfolio’s (not-)permitted to be marketed to children the WHO-model was applied. The WHO-model determines per product category whether products should be (not-)permitted to be marketed to children. An overview of the 17 product categories included in the WHO-model can be found in Supplementary file 3. While a threshold for nutrients of concern determines if a product could be permitted to be marketed to children for most product categories, some categories are entirely permitted (such as ‘*Fresh and frozen meat, poultry, fish and similar*’ and ‘*Fresh and frozen fruit, vegetables and legumes*’) or not-permitted to be marketed to children (such as ‘*Chocolate and sugar confectionery, energy bars, and sweet toppings and desserts*’; ‘*Cakes, sweet biscuits and pastries, other sweet bakery wares, and dry mixes for making such*’; ‘*Juices*’; ‘*Energy drinks’* and ‘*Edible ices’*) [31]. From a public health perspective it would be expected that companies with a higher proportion of products not-permitted to be marketed to children would have stronger commitments in place to reduce such practices.

To specifically evaluate the products promoted by supermarkets, food promotions in the flyers of the six biggest supermarkets in France were collected online from the weekly/two-weekly circulars over a six-month period (October 2019 – March 2020). All promotions were entered into a database and manually classified according to the NOVA-classification and the 17 food categories of the WHO-model (Supplementary file 3). Per product the following information was recorded: product- and brand name, type of promotional character, the level of discount, type of incentive offer, if the product was a fresh fruit or vegetable, whether the product was a fresh meat or fish product and the Nutri-Score [38]. The proportion of promotions for ultra-processed foods, foods with promotional characters, incentive offers or discounts and the proportion of promotions for fresh fruits and vegetables were calculated. Data were analysed separately per supermarket.

#### The relationship between commitments and practices

Correlations (𝝆-values) between commitments and practices were calculated applying the Spearman’s rank correlation coefficient, a non-parametric test that measures the direction and strength of a monotonic association between two variables. 𝝆-values range from − 1, indicating a perfect negative correlation between two variables to + 1, indicating a perfect positive correlation between variables. Correlations were calculated between commitments made within the domain ‘Product formulation’ and the proportion of products within the portfolio with Nutri-Score A and B and D and E. Correlations between the domain ‘Product formulation’ and the proportion of ultra-processed products were also calculated. Lastly, correlations between commitments within the domain ‘Product and brand promotion’ and the proportion of products not-permitted to be marketed to children according to the WHO-model were assessed.

𝝆 -values between 0.5 and 1 as well as between − 0.5 and − 1 were considered to represent a moderate to strong correlation. *P*-values < 0.05 were considered statistically significant. All analyses were performed using Microsoft Excel and SAS 9.4 (Cary, USA, 2018).

## Results

### Nutrition-related commitments

**Fig. 1:**
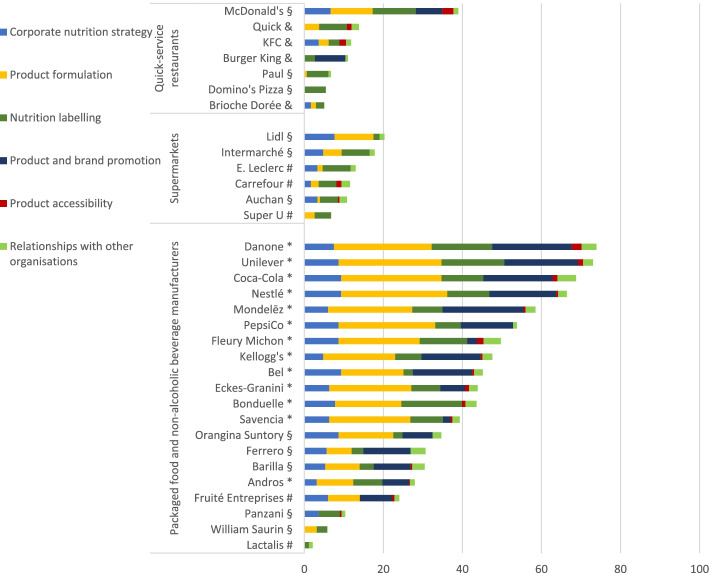
Business Impact Assessment on Obesity and Population Nutrition (BIA-Obesity), France 2020 – Overall and domain-specific scores for quick-service restaurants, supermarkets and packaged food and non-alcoholic beverage manufacturers. * Full engagement with the process (*N =* 13); # Declined participation (*N =* 5); § Accepted participation, but contributions not received in time (*N =* 11); & Not able to contact the company (*N =* 4); For #, § and &: Assessment of commitments was based on publicly available information only

Out of the 33 selected food companies, 13 verified and completed the publicly available information, 11 accepted participation but did not provide feedback in time, five declined participation and four companies were unreachable (Fig. 1).

French food companies demonstrated some commitment to improving population nutrition, but much stronger action is needed across sectors and across BIA-Obesity policy domains. The overall scores ranged from 2% (Lactalis) up to 74% (Danone) with a median overall score of 28% (IQ*R =* 34). The best performing domain was ‘Corporate nutrition strategy’ (median score = 53%, range = 0-93%, IQR = 60) while the worst performing domain was ‘Product accessibility’ (median score = 6%, range = − 10-50%, IQR = 10). Packaged food and beverage manufacturers had substantially more transparent, comprehensive and specific commitments in place with a median overall BIA-Obesity scores of 44% (range = 2-74%, IQR = 25) compared to 12% for supermarkets (range = 7-20%, IQR = 6) and 11% for quick-service restaurants (range = 5-39%, IQR = 7). Domain-specific scores were also lower for quick-service restaurants and supermarkets (considered as both retailer and packaged food and non-alcoholic manufacturer) than for packaged food and beverage manufacturers. In particular the median score for both the domains ‘Product and brand promotion’ and ‘Product accessibility’ was 0 for quick-service restaurants and supermarkets.

Scores per BIA-Obesity domain and per company are presented in Table 3. For the 13 food companies that participated (response rate = 39%), the median overall BIA-Obesity score significantly increased from 38% (scoring based on public information only) to 50% (scoring after full participation) (*p* < 0.001). The 20 companies that did not participate and engage with the BIA-Obesity process obtained significantly lower median overall BIA-Obesity scores (12%, IQR = 14) compared to the 13 companies that did engage with the process (median = 50%, IQR = 23) (*p* < 0.05).

**Table 3:**
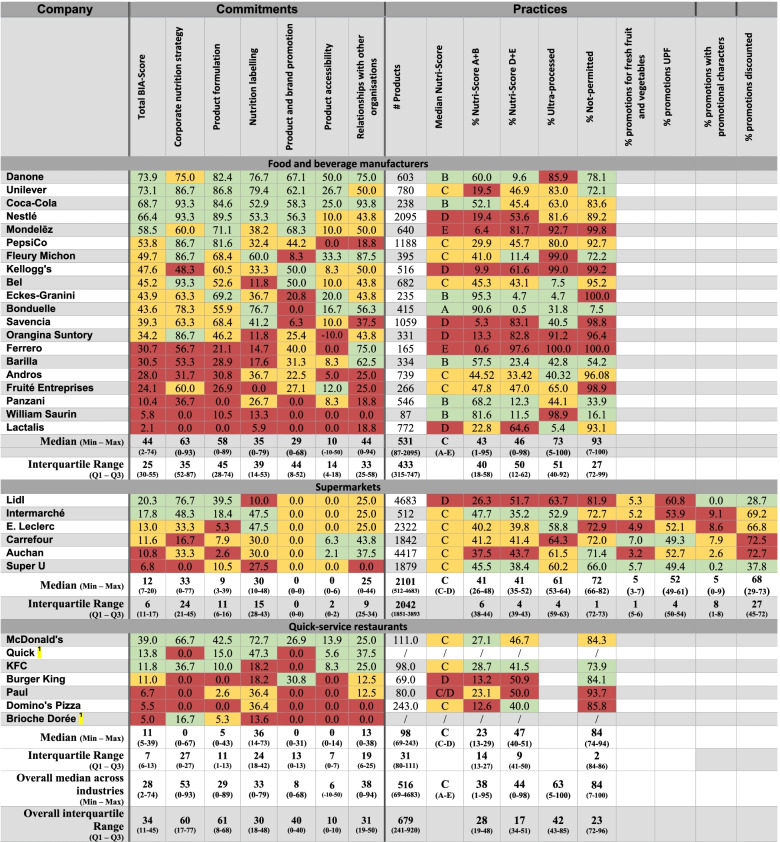
An overview of the final French ‘Business Impact assessment on Obesity and Population Nutrition’ (BIA-Obesity) scores for commitments and practices per company. Data are sorted by descending total BIA-Obesity score per food industry (food and beverage manufacturers, supermarkets and quick-service restaurants). Green indicates a score within the top third of companies per food industry and red indicates a score within the lowest third of companies per food industry. Yellow indicates the companies in between. / indicates that no data were available

On the website of Brioche Dorée and Quick no nutritional information was available per 100 g and portion sizes were not defined. As a result the product portfolios of Brioche Dorée and Quick could not be analysed. This is indicated in the table with a ‘/’.

Within the ‘Corporate nutrition strategy’ domain seven out of the 33 companies had no commitments in place. Packaged food and beverage companies (median = 63%) performed better than supermarkets (median = 33%) and quick-service restaurants (median = 0%) for this domain. Some companies recognized both national (i.e. Nutri-Score) as well as international (i.e. The United Nations Sustainable Development Goals or the World Health Organization global NCD action plan) priorities within their corporate nutrition strategy.

Others published annual national reports detailing their progress against objectives and targets. The lowest performing companies made little or no mention of nutrition-related issues and did not identify population nutrition as a clear priority focus area.

Within the ‘Product accessibility’ domain only a limited number of companies made commitments to address the accessibility of healthy compared to ‘less healthy’ products (12 out of the 33 companies). Packaged food and beverage manufacturers had the highest median score (10%) while supermarkets and quick-service restaurants had a median score of 0%, lacking all commitments regarding best practice actions in this domain, such as confectionary free checkouts for supermarkets or commitments to limit supersizing among quick-service restaurants. The implementation of taxes on some unhealthy food products was supported by three companies and opposed by seven. Supermarkets neither opposed or supported the implementation of such taxes.

The median score within the domain ‘Product formulation’ was 29% (0-89%; IQR = 61) with food and beverage manufacturers scoring the highest (58%) followed by supermarkets (9%) and quick-service restaurants (5%). Nestlé obtained the highest score while four companies made no commitments in this area (Lactalis, Panzani, Burger King and Domino’s Pizza). 11 out of 20 food and beverage manufacturers and two supermarkets had targets in relation to reducing the sodium content, while 14 out of 20 food and beverage manufacturers and two supermarkets had targets in relation to reducing the added sugar content. Eight out of 20 food and beverage manufacturers and one out of five supermarkets had targets in relation to reducing portion sizes. Only one out of seven quick-service restaurants had such targets. Three out of the 20 food and beverage manufacturers applied the Nutri-Score to guide reformulation. This was not the case for any quick-service restaurants or supermarkets.

The domain ‘Nutrition labelling’ obtained a median score of 33% (0-79%; IQR = 30). All companies apart from one (Fruité Entreprises) made commitments within this area. When comparing food industries, food and beverage manufacturers (median = 35%) and quick-service restaurants (median = 36%) performed better than supermarkets (median = 30%). The top performer in this domain (Unilever) publicly committed to link the use of nutrition and health claims to the healthiness of products as determined by their own classification system. Two additional companies had a similar commitment in place, but this was not publicly available. 12 out of 20 packaged food and beverage manufacturers and all six supermarkets committed to implement the government-endorsed Nutri-Score on their (own-brand) products. All quick-service restaurants provided nutritional information about products online to some extent, although sometimes only per serving (without indication of portion size) instead of per 100 g. In addition, four out of seven quick-service restaurants committed to labelling their menu boards in-store.

The domain ‘Product and brand promotion’ obtained a median score of 8% (range = 0-68%; IQR = 40) and was the second worst scoring BIA-Obesity domain in France. Food and beverage manufacturers obtained a median score of 29%, while supermarkets and quick-service restaurants obtained a median score of 0%. 15 out of all 33 companies had no commitments within this domain, including all six supermarkets and five out of the seven quick-service restaurants. None of the selected companies developed marketing policies for children up to the age of 18 years and only three packaged food and beverage manufacturers committed not to sponsor children’s sporting, cultural or other activities using unhealthy foods and brands.

Lastly, the median score for the domain ‘Relationships with other organisations’ was 38% (range = 0-94%; IQR = 31). Only four companies did not have any commitments within this domain (William Saurin, Brioche Dorée, Domino’s Pizza and Super U). Median scores per food industry ranged from 13% for quick-service restaurants up to 25% for supermarkets and 44% for food and beverage manufacturers.

### Practices

The performance results per indicator and per company are shown in Table 3.

#### Product formulation

Across all selected food companies, the proportion of portfolios consisting of A and B Nutri-Score products ranged from 0.6% for Ferrero to 95% for Eckes-Granini (median = 38%; IQR = 28). One food and beverage company had a median Nutri-Score A (Bonduelle) across its entire portfolio while two companies had a median Nutri-Score E (Ferrero and Mondelēz). The product portfolios of supermarket own-brand products and quick-service restaurants all had a median Nutri-Score C apart from one supermarket (Lidl) and two quick-service restaurants (Burger King and Paul). The proportion of products within portfolios with Nutri-Score A and B ranged from 1 to 95% for food and beverage manufacturers (median = 43%; IQR = 40), 26 to 48% for supermarkets (median = 41%; IQR = 6) and from 13 to 29% for quick-service restaurants (median = 23%; IQR = 14). The median proportion of ultra-processed food products within portfolios of selected food and beverage manufacturers was 73% (range = 5-100%; IQR = 51). For supermarkets this was 61% (range = 53-64%; IQR = 4).

#### Product and brand promotion

According to the WHO-model, the median proportion of products within portfolios across food and beverage manufacturers not-permitted to be marketed to children was 93% (ranging from 7% for Bonduelle to 100% for Ferrero and Eckes-Granini; IQR = 27). For quick-service restaurants this was 84% (range = 74-94; IQR = 2), and for supermarkets this was 72% (range = 66-82%; IQR = 1).

For the food promotions in the supermarket flyers, it was found that promotions were mostly for ultra-processed foods (median = 52%; IQR = 4). Nonetheless, considerable variation was observed between the different supermarkets with the proportion of promotions for ultra-processed foods ranging from 49% (Carrefour and Super U) up to 61% (Lidl) of all promotions. Across the entire circular, Carrefour most frequently promoted fresh fruits and vegetables (7% of all promotions) and Auchan least frequently (3% of all promotions). Throughout the flyers only around 5% (range = 0-9%; IQR = 8) of promotions had promotional characters while 68% of products were discounted (range = 29-73%; IQR = 27) (Table 3).

### The association between commitments and practices

Table 4 shows that no significant correlations were observed between commitments within the domains ‘Product formulation’ and ‘Product and brand promotion’ and respective performance indicators. As no supermarkets and only two out of five quick-service restaurants made commitments to limit marketing to children within the domain ‘Product and brand promotion’, no correlations with practices, as assessed by the WHO-model, could be calculated for these food industries (Table 4). As none of the French supermarkets had a commitment in place to have a minimum proportion of products promoted in their regular flyers to be healthier products, no correlation could be calculated between commitments and the healthiness of products promoted in supermarket flyers.

**Table 4:** The correlations calculated between the commitments within the domains ‘Product formulation’ and ‘Product and brand promotion’ and the respective performance indicators (% Nutri-Score A + B; % Nutri-Score D + E; % Ultra-Processed and % Not-permitted to be marketed to children according to WHO)

Correlation	𝝆 *(Spearman’s Rank Correlation Coefficient)*	*P*-value
Food and beverage manufacturers		
Product formulation and % Nutri-Score A + B	−0.150	0.529
Product formulation and % Nutri-Score D + E	−0.043	0.858
Product formulation and % ultra-processed	0.123	0.605
Product formulation and % not-permitted	0.023	0.922
Product and brand promotion and % not-permitted	0.252	0.285
Supermarkets		
Product formulation and % Nutri-Score A + B	0.143	0.787
Product formulation and % Nutri-Score D + E	−0.086	0.872
Product formulation and % ultra-processed	−0.029	0.957
Product formulation and % not-permitted	0.429	0.397
Quick-service restaurants		
Product formulation and % Nutri-Score A + B	−0.564	0.322
Product formulation and % Nutri-Score D + E	0.051	0.935
Product formulation and % not-permitted	−0.410	0.493

Visualizing the results shown in Table 4, it can be observed in Table 3 that food companies within the top third for commitments within the domain of ‘Product formulation’ don’t necessarily have the healthiest portfolios as determined by the Nutri-Score and NOVA-classification. On the contrary, there are companies within the lowest third for commitments that still have among the heathiest portfolios. The same can be observed for commitments and practices within the domain ‘Product and brand promotion’

## Discussion

This study quantitatively assessed for the first time the commitments and practices related to obesity prevention and population nutrition of the major food companies in France. The findings showed a large variation between companies based on the overall scores for the transparency, comprehensiveness and specificity of commitments as well as the performance indicators. Overall BIA-Obesity scores ranged from 2 to 74% (median = 28%). The median overall score was 11% for quick-service restaurants, 12% for supermarkets and 44% for packaged food and non-alcoholic beverage manufacturers. The best performing domain was ‘Corporate nutrition strategy’ while the worst performing domain was ‘Product accessibility’. The performance indicators indicated that the majority of portfolios consisted of ultra-processed foods (63%) and products not-permitted to be marketed to children according to WHO (84%). Only a limited proportion of the promotions in supermarket flyers was for fresh fruits and vegetables while more than half of the promotions were for ultra-processed foods. Performance metrics relating to food formulation and marketing were not associated with the overall BIA-Obesity score on commitments.

The overall BIA-Obesity scores in France were lower than the scores obtained in previous studies in Australia, New Zealand and Belgium, but higher than the scores in Malaysia. This observation matches the response rates which were higher than in Malaysia, but lower than in the other countries where companies had the opportunity to complete and verify the data [13–15, 17]. BIA-scores and response rates are presented in Supplementary file 4. As previous research has shown that the BIA-Obesity scores significantly increase for companies that engage with the process [13, 14, 17], the lower response rate in France might be able to explain the lower BIA-Obesity scores. Since this is the first assessment in France, it is anticipated that more companies will engage with future assessments. In France it was also observed that quick-service restaurants and supermarkets scored notably lower than packaged food and non-alcoholic beverage manufacturers. This difference might, in part, be attributable to the fact that among the latter some companies verified and completed the data while for all quick-service restaurants and supermarkets the assessment was based solely on publicly available data as the companies within these industries could not be reached, declined participation or did not provide feedback in time (indicated in Fig. 1). However, BIA-Obesity scores in Australia and Malaysia followed a similar trend [14, 15], suggesting that the observed difference between food industries might not solely be attributable to the difference in response rates. As both quick-service restaurants and supermarkets are in direct contact with consumers, the domain ‘Product accessibility’ has a higher weighting (weighting of 20%) than it has among packaged food and non-alcoholic beverage manufacturers (weighting of 5%; Supplementary file 2) [18]. Potentially not by coincidence, this domain was also observed as the worst performing BIA-Obesity domain. Consequently, this difference in weighting might also contribute to the lower overall BIA-Obesity scores of quick-service restaurants and supermarkets.

Across all abovementioned countries, ‘Corporate nutrition strategy’ was the best performing BIA-Obesity domain and ‘Product accessibility’ the worst [13–15, 17], findings similar to what was observed at global level by the ‘Access To Nutrition Index’ (ATNI) in 2018 and 2021 [39, 40]. The ATNI benchmarks food company commitments and practices in a similar way to the BIA-Obesity, but does this at global level for only food and beverage manufacturers. As it is a global assessment, the ATNI looks at both over- and undernutrition, something that is not the case for BIA-Obesity [18, 19, 41]. France however scored notably lower in the domain ‘Product and brand promotion’ compared to other countries. Most likely this can be attributed to the lack of a (voluntary) code to restrict marketing to children in France, something that is in place in Belgium, New Zealand and Australia [24–26].

Similar across all studies and countries however, company commitments fell short of recommended best practices. To improve commitments companies should use an official nutrient profiling system (such as the Nutri-Score) to guide reformulation of products and ensure time-bound reduction of nutrients of concern such as salt, sugar, trans-fat, saturated fat and the energy content. Furthermore, it is recommended for companies to limit marketing to all children below the age of 18 to products that meet the WHO-model nutrition criteria. Specifically for packaged food and beverage manufacturers it is advised to limit the use of nutrition and health claims to products that are healthy according to an official nutrient profiling system such as the Nutri-Score. For quick-service restaurants it would be desirable to make nutritional information available on menus. Preferably, quick-service restaurants would also commit to not open new outlets within walking distance of schools. Finally, French supermarkets need to step up their commitments in the areas of ‘Product and brand promotion’ and ‘Product accessibility’ as none of the selected supermarkets made commitments to limit marketing to children, to limit the in-store promotion of less healthy products or to increase the accessibility of healthier products compared to less healthy alternatives. Underlying the importance of strengthening the commitments lies the assumption that stronger commitments will translate into improved practices and performance. Similar to earlier research however, this study found no relationship between voluntary commitments and healthier product portfolios [16, 42]. More importantly, earlier research pointed towards the importance of being cautious with voluntary company commitments as these might help to legitimize and advertise the food industry’s role in improving population health without any assurance that company practices go beyond business as usual [43, 44]. Providing the food industry with an official communication platform through public-private partnerships might even undermine public health policies. For example, such platforms can provide companies with the opportunity to influence the public discourse regarding health (e.g. focus on individual responsibility and freedom of choice [45]) and frame public health problems and potential solutions [43, 46]. Consequently it is important to monitor the relationship between company commitments and practices, and ensure that appropriate performance metrics are used to assess how company commitments translate into practice. Government regulation remains primordial to ensure better company practices and healthier food environments [47].

When comparing performance indicators across Belgium and France it was observed that overall median product portfolios have a higher proportion of products with Nutri-Score A and B [17]. This could potentially be explained by the fact that the Nutri-Score became the government endorsed FOP labelling system in France in 2017 [21] while it was only introduced in Belgium in 2019 [22, 48]. Also the proportion of product portfolios consisting of ultra-processed products was slightly lower in France (63%) than what was observed in Belgium (75%) [17]. This observation is in line with previous research that found a significant higher household availability of ultra-processed food products in Belgium than in France [7]. Nonetheless, such numbers are of concern as a recent study in France found a probable association between the consumption of ultra-processed foods and a higher mortality risk [9]. This association is however merely part of the growing body of literature highlighting the risks of ultra-processed food consumption [6–8]. Eventually, the proportion of products permitted to be marketed to children was similar across these neighbouring countries, standing at 16% in France and 19% in Belgium [17]. These findings are also similar to those of ATNI in 2018 and 2021 that found that only 14 and 9%, respectively, of product portfolios of the major multinational companies consisted of products permitted to be marketed to children according to WHO [39, 40]. The lower percentage in 2021 might be because the latest ATNI study used the regional WHO nutrient profile models instead of the European WHO-Model that was used in the 2018 ATNI and the abovementioned BIA-Obesity studies [17, 39, 40].

An important strength of this study is that it allows for a first intra-European country comparison of BIA-Obesity data in regards of both commitments and performance. Nonetheless, towards the future a more in-depth analysis comparing BIA-Obesity data across a wider range of European countries would be recommended, especially including countries from different European regions. An important limitation of this French BIA-Obesity study is the low response rate of company representatives (39%). As less than half of the companies verified and completed the publicly available data it might be that in reality the BIA-Obesity scores are higher than what was observed in the study. Even so, it is expected that response rates will increase during future iterations. Concerning the performance data, Open Food Facts data had to be used. Consequently, it cannot be guaranteed that all products present on the market in 2018 were included in the study. Moreover, some level of data duplication might be possible. Even though it was ensured that each barcode appeared only once in the database, products that changed barcode throughout the year or had wrong barcodes assigned within the Open Food Facts database might be accounted for multiple times. Another limitation is the fact that performance indicators were not able to capture changes overtime in the healthiness of product portfolios of selected companies potentially resulting from the commitments in place. To overcome this limitation, it is recommended for following studies to assess the associations between commitments in place and the changes of performance indicators over time. Within the current iteration of the BIA-Obesity the proportion of products within company portfolios (not)permitted to be marketed to children according to the WHO model was assessed. However, such performance metric does not capture to what extent such not-permitted products are in practice marketed to children across various media and settings by food companies. Consequently it is recommended for future iterations to assess the extent and nature of not-permitted food and beverage advertisements targeted to children in (non-)broadcast media. Eventually, due to data availability and time constraints, this study did not capture practices related to corporate political activities (such as lobbying or research funding) that may affect food policies. Including performance data on such practices might however be able to partially explain why no association can be found between commitments and practices.

## Conclusions

In conclusion, although French food companies have taken a few steps as part of a societal response to unhealthy diets and obesity, there is a much greater role for them to play. The overall and domain-specific BIA-Obesity scores showed that there is a lot of room for food companies across all four industries to improve the comprehensiveness, specificity and transparency of their nutrition-related commitments, as well as their practices related to population nutrition, in particular ‘Product reformulation’ and ‘Product and brand promotion’. The next iterations of the BIA-Obesity should include a wider list of performance metrics of companies in relation to product formulation, labelling, promotion and accessibility. In view of these results, it is clear that stronger government regulations on food environments will be essential to achieve the goals of the World Health Organization action plan on chronic diseases as well as the Sustainable Development Goals.

## Supplementary Information

**Additional file 1: Supplementary file 1.** Examples of how publicly available commitments were collected and scored according to the Business Impact Assessment on Obesity and Population Level Nutrition (BIA-Obesity) tool. **Supplementary file 2.** Weighting per ‘Business Impact Assessment on Obesity and Population Nutrition’ (BIA-Obesity) domain and food industry. **Supplementary file 3.** The 17 food categories included in the World Health Organisation Regional Office for Europe nutrient profile model (WHO-model) [31]. **Supplementary file 4.** Overall median ‘Business Impact assessment on Obesity and Population Nutrition’ (BIA-Obesity) scores across countries where data were collected for food and beverage manufacturers, supermarkets and quick-service restaurants and companies had the opportunity to verify and complete the publicly available data [13–15].

## Data Availability

The datasets used and/or analysed during the current study are available from the corresponding author on reasonable request.

## Abbreviations

ATNI: Access To Nutrition Index
BIA-Obesity: Business Impact Assessment on Obesity and population-level nutrition
BMI: Body Mass Index
INFORMAS: International Network for Food and Obesity/Non-communicable Disease Research, Monitoring and Action Support
GNPD: Global New Products Database
NCDs: Non-Communicable Diseases
SMART: Specific, Measurable, Achievable, Relevant and Time bound
WHO-model: World Health Organisation Regional Office for Europe nutrient profile model
