# Supplementary material for: Benchmarking the nutrition-related commitments and practices of major French food companies
Source: BMC Public Health. 2022 Jul 28;22:1435. doi: 10.1186/s12889-022-13780-y (PMC9330679; doi:10.1186/s12889-022-13780-y)
Supplement: Supplementary file 1 — Additional file 1: Supplementary file 1. Examples of how publicly available commitments were collected and scored according to the Business Impact Assessment on Obesity and Population Level Nutrition (BIA-Obesity) tool. Supplementary file 2. Weighting per ‘Business Impact Assessment on Obesity and Population Nutrition’ (BIA-Obesity) domain and food industry. Supplementary file 3. The 17 food categories included in the World Health Organisation Regional Office for Europe nutrient profile model (WHO-model) [31]. Supplementary file 4. Overall median ‘Business Impact assessment on Obesity and Population Nutrition’ (BIA-Obesity) scores across countries where data were collected for food and beverage manufacturers, supermarkets and quick-service restaurants and companies had the opportunity to verify and complete the publicly available data [13–15]. [file 12889_2022_13780_MOESM1_ESM.docx]

**Supplementary files**

**Supplementary file 1:** Examples of how publicly available commitments were collected and scored according to the Business Impact Assessment on Obesity and Population Level Nutrition (BIA-Obesity) tool.

| Domain | Indicator | Policy content | Scoring criteria | Score |
| --- | --- | --- | --- | --- |
| Corporate nutrition strategy | *Does the company have an overarching commitment to improving population nutrition and health articulated in strategic documents (e.g., corporate strategy document, corporate responsibility reports)?* | “According to the WHO, it is important to limit the intake of sugar, salt and fat in the fight against welfare diseases (obesity and diet-related diseases). We therefore strive to limit the content of these nutrients in our products.”  – Friesland Campina (49) | 10: Yes, a specific national-level (country-specific) commitment to improving population nutrition and health, publicly available in strategic documents  7.5: Yes, a specific global- or European level (not country -specific) commitment to improving population nutrition and health, publicly available in strategic documents  5: Yes, a national-, European- or global- level commitment, but not publicly-available, OR general reference to nutrition and health as part of general corporate strategy  0: No clear commitments to improving population nutrition and health | *7.5* |
| Product formulation | *Has the company set a target/targets or provided detailed evidence of having taken significant action to reduce/reach lower levels of added sugars, and is it applicable to the country in question?* | “Thanks to this continued commitment, we want to achieve a 17.7% reduction in the average sugar content of our beverages by 2020 compared to 2010. Calorie reduction per litre through reduced sugar content”  – Coca-Cola (50,51) | 10: Set SMART targets or provided detailed evidence of having taken significant action in all key categories/subcategories, published  5: Targets (not necessarily SMART) set or taken significant action in some key products/sub-categories / not published  2.5: General commitment to reducing use of added sugars in products (vague or global level only), published or disclosed to INFORMAS team  0: No target / no information | *10* |
| Nutrition labelling | *Does the company have a published commitment to rolling out the government-endorsed Nutri-Score?* | “McCain is committed to the implementation of the Nutri-Score. Further explanation about the Nutri-Score can be found on McCain's website.”  - McCain (52) | 10: Yes, with implementation plan across all product categories (published or unpublished)  7.5: Yes, with implementation plan across a selection of product categories (published or unpublished)  5: Yes, but with no specific implementation plan (published or unpublished)  0: No | *10* |
| Product and brand promotion | *Does the company have an explicit policy to reduce the exposure of children to unhealthy food marketing on broadcast media (TV, radio)?*  *(Note: check if the company supports the Belgian Pledge. If yes and no other comments, then Belgian pledge is scored)* | Signatory to the Belgian Pledge, but not mentioned on company website.  - Schweppes, GB Foods, Imperial Meat Products, Lotus Bakeries, Aldi, Carrefour, Colruyt, Lidl (24) | 10: Yes, national policy and noted on company website / annual reports  7.5: Yes, global policy and noted on company website / annual reports  5: Yes, national policy but not noted on company website / annual reports OR national policy and noted on industry association website  2.5: Yes, global policy but not noted on company website / annual reports  0: No policy/ no information available to the research team | *5* |
| Product accessibility | *Does the company publish its policy position (in relation to government policy) on fiscal policies to make healthier foods relatively cheaper and unhealthy foods relatively more expensive?* | “Fiscal instruments related to nutrition: In general, alongside healthy lifestyle education and more physical activity, we believe reformulating some of our products, widening and improving the nutritional composition of our portfolio, providing information to help consumers make healthier choices through nutritional labeling and following self-imposed marketing and advertising restrictions to protect children are far more effective ways we can help people achieve their dietary goals. Public policy measures should be designed in such a way as to encourage companies to be active in these areas.”  - PepsiCo (53) | 10: Yes, on own website  5: Yes, on industry association website  0: Not publicly available | *10* |
| Relationships with other organisations | *Does the company publish details of the nutrition education / healthy diet oriented programs it funds or supports?* | “One of the core activities of Cultureghem supported by Nestlé is KOOKMET. This is an information and awareness programme on nutrition and the importance of vegetables in the diet. Children from neighbouring schools, their parents or pupils from other regions in Belgium learn to use local, simple ingredients within a limited budget. Buying ingredients at the local market, learning to cook together, being aware of the value of the prepared dishes (both in terms of money and nutrition), having fun, making contacts and having conversations and then eating together or with strangers.”  - Nestlé (54) | 10: Yes, information on national-level activity is publicly available (website or document) in a consolidated and cumulative form OR active declaration/policy stating no activity in this area (either publicly available or disclosed to INFORMAS team)  5: Yes, information is available, but is not consolidated and easy to locate OR information is available at the global level only OR comprehensive information about their activities in the area provided to the project team  0: No information available / provided | *10* |

**Supplementary file 2:** Weighting per ‘Business Impact Assessment on Obesity and Population Nutrition’ (BIA-Obesity) domain and food industry (Belgium, 2020).

| **BIA-Obesity domains** | **Packaged food and non-alcoholic beverage manufacturers** | **Quick-service restaurants** | **Supermarkets** |
| --- | --- | --- | --- |
| **Corporate nutrition strategy** | 10 | 10 | 10 |
| **Product formulation** | 30 | 25 | 25 |
| **Nutrition labelling** | 20 | 15 | 15 |
| **Product and brand promotion** | 30 | 25 | 25 |
| **Product accessibility** | 5 | 20 | 20 |
| **Relationships with other organisations** | 5 | 5 | 5 |
| **TOTAL** | **100** | **100** | **100** |

**Supplementary file 3:** The 17 food categories included in the World Health Organisation Regional Office for Europe nutrient profile model (WHO-model) (31).

| **Group** | **Name** |
| --- | --- |
| **1** | Chocolate and sugar confectionery, energy bars, and sweet toppings and desserts |
| **2** | Cakes, sweet biscuits and pastries; other sweet bakery wares, and dry mixes for making such |
| **3** | Savoury snacks |
| **4** | Beverages |
| **4A** | a) Juices |
| **4B** | b) Milk drinks |
| **4C** | c) Energy drinks (often contain o.a. guarana, taurine, glucuronolactone and vitamins) |
| **4D** | d) Other beverages (Soft drinks, sweetend beverages) |
| **5** | Edible ices |
| **6** | Breakfast cereals |
| **7** | Yoghurts, sour milk, cream and other similar foods |
| **8** | Cheese |
| **9** | Ready-made and convenience foods and composite dishes |
| **10** | Butter and other fats and oils |
| **11** | Bread, bread products and crisp breads |
| **12** | Fresh or dried pasta, rice and grains |
| **13** | Fresh and frozen meat, poultry, fish and similar +eggs |
| **14** | Processed meat, poultry, fish and similar |
| **15** | Fresh and frozen fruit, vegetables and legumes |
| **16** | Processed fruit, vegetables and legumes |
| **17** | Sauces, dips and dressings |

**Supplementary file 4:** Overall median ‘Business Impact assessment on Obesity and Population Nutrition’ (BIA-Obesity) scores across countries where data were collected for food and beverage manufacturers, supermarkets and quick-service restaurants and companies had the opportunity to verify and complete the publicly available data (13–15).

| Country | Total Score | Corporate nutrition strategy | Product formulation | Nutrition labelling | Product and brand promotion | Product accessibility | Relationships with other organisations | Response rate |
| --- | --- | --- | --- | --- | --- | --- | --- | --- |
| Australia  (2018) | 41 | 55 | 40 | 54 | 36 | 5 | 44 | 47 % |
| New Zealand  (2017) | 38 | 55 | 34 | 47 | 35 | 0 | 38 | 48 % |
| Belgium  (2019-2020) | 35 | 57 | 37 | 32 | 36 | 8 | 33 | 56 % |
| Malaysia  (2014-2017) | 11 | 28 | 8 | 15 | 0 | 4 | 25 | 18% |
